# miR-142 induces accumulation of reactive oxygen species (ROS) by inhibiting pexophagy in aged bone marrow mesenchymal stem cells

**DOI:** 10.1038/s41598-020-60346-2

**Published:** 2020-02-28

**Authors:** Kei Houri, Tatsufumi Mori, Yuta Onodera, Takatoshi Tsujimoto, Toshiyuki Takehara, Shinichi Nakao, Takeshi Teramura, Kanji Fukuda

**Affiliations:** 1 Department of Anesthesiology, Kindai University Faculty of Medicine, Osaka, Japan; 2 Kindai University Life Science Research Institute, Kindai University, Osaka, Japan; 3 Division of Cell Biology for Regenerative Medicine, Institute of Advanced Clinical Medicine, Kindai University Faculty of Medicine, Osaka, Japan

**Keywords:** Pexophagy, Ageing, Mesenchymal stem cells

## Abstract

Elevation of the levels of reactive oxygen species (ROS) is a major tissue-degenerative phenomenon involved in aging and aging-related diseases. The detailed mechanisms underlying aging-related ROS generation remain unclear. Presently, the expression of microRNA (miR)-142-5p was significantly upregulated in bone marrow mesenchymal stem cells (BMMSCs) of aged mice. Overexpression of miR-142 and subsequent observation revealed that miR-142 involved ROS accumulation through the disruption of selective autophagy for peroxisomes (pexophagy). Mechanistically, attenuation of acetyltransferase Ep300 triggered the upregulation of miR-142 in aged BMMSCs, and miR-142 targeted endothelial PAS domain protein 1 (Epas1) was identified as a regulatory protein of pexophagy. These findings support a novel molecular mechanism relating aging-associated ROS generation and organelle degradation in BMMSCs, and suggest a potential therapeutic target for aging-associated disorders that are accompanied by stem cell degeneration.

## Introduction

Aging worsens functions of human tissues and organs at multiple levels, causing a gradual reduction in the ability to resist stress, damage, and various related diseases. Cellular senescence is considered an important aging hallmark and the direct reason for the above mentioned changes^1,2^. In recent years, accumulating evidence has indicated that reactive oxygen species (ROS), which include superoxide anion and hydroxyl radicals, generated from both intrinsic and extrinsic events induce cell damage and senescence during aging. There are numerous studies, which report that ROS and oxidative damage increase with age^3,4^, that reducing oxidative damage extends the lifespan of various model organisms, and increased production of ROS shortens their lifespan^5^. ROS contribute to cellular senescence onset and progression by damaging mitochondrial DNA (mtDNA) and modifying the telomerase reverse transcriptase (TERT) enzyme^6^, histones, and DNA by acting in interconnected epigenetic phases^7,8^. Furthermore, high ROS levels provoke p53 activation, which induces p53-mediated apoptosis and cell senescence^9^. Although all cells in an organism can be affected by the accumulation of ROS, the effects of ROS on stem cells are particularly important for understanding the processes of aging and its related diseases^10,11^. Accumulation of oxidative damage in stem cells can lead to loss of stemness, cell transformation, tumorigenesis, or tissue injury^11^. Thus, elucidating the molecular mechanisms underlying ROS accumulation in stem cells is important to develop therapies for inhibiting the underlying cause of aging-related tissue dysfunction or diseases.

Degenerated cellular organelles are a major source of ROS. Oxygen is consumed in various metabolic reactions in different intracellular locations, with mitochondria, ER, and peroxisomes being the major sites; thus, dysfunction of these organelles directly leads to the generation of a large amount of ROS^12^. Among these, the peroxisome is a very important source of ROS, which is mainly produced through metabolic pathways, including fatty acid β-oxidation, photorespiration, nucleic acid, and polyamine catabolism^13–15^. It has been estimated that about 35% of all H_2_O_2_ formed in the rat liver is derived from its peroxisomes. The number, morphology, and size of peroxisomes are dynamically regulated in response to environmental and developmental cues^16^. Selective autophagy for peroxisomes, called pexophagy, is a quality control mechanism to maintain the proper function of peroxisomes^15^. However, the decline of autophagic activity and accumulation of damaged macromolecules and organelles are well known characteristics of aged cells^17–19^. Importantly, increases in peroxisome number,^20^ along with reduction of peroxisomal enzymes^20,21^ during aging, has also been reported. Although partial mechanisms inducing the decreased expression of genes associated with autophagy have been discovered^22^, the mechanisms underlying the impairment of autophagic function by aging remain poorly understood.

Recently, short strand noncoding RNAs called miRNAs have been found to be involved in the regulation of autophagy^23^. During aging, it has been reported that certain miRNAs are involved in aging-associated degenerative changes, including inhibition of autophagy and promotion of ROS generation^24–26^.

Here we identified *miR-142-5p* (*miR-142*) as a prominent miRNA in aged BMMSCs and found that *miR-142* induced increasing numbers of peroxisomes and cellular ROS levels by inhibiting pexophagy through the suppression of Epas1 expression.

## Results

**Figure 1:**
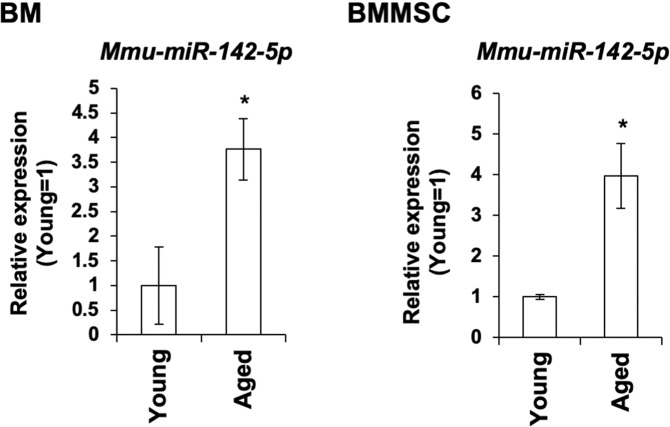
m*iR-142-5p* (*miR-142*) expression is upregulated in aged BMMSCs. qRT-PCR of *miR-142* in BM tissue (N = 6) and *miR-142* in PDGFRα/Sca1-double positive (PαS) BMMSCs of young and aged mice (N = 6). Asterisk represents a significant difference compared with the BM or PαS of young mice at *P* < 0.05.

To identify miRNAs upregulated in aged BMMSCs, we performed miRNA-sequencing for PαS-double positive cells collected from the bone-marrow (BM) of young and aged mice. It was revealed that 32 miRNAs exhibited a log2 fold change of >3 in the aged BMMSCs. Among these, we chose miRNAs showing a mean value of relative expression >1,000 and focused on *miR-142* since it showed the largest difference in expression levels between young and aged BMMSCs (Table S1), and its expression level was higher than ubiquitously expressed miRNA in undifferentiated BMMSCs such as *miR-125b*^27^, *miR-204*^28^, and *let7c*^29^. Quantitative RT-PCR (qRT-PCR) showed the upregulation of *miR-142* in the BM and in BMMSCs collected from aged mice (Figs. 1 and S1).

**Figure 2:**
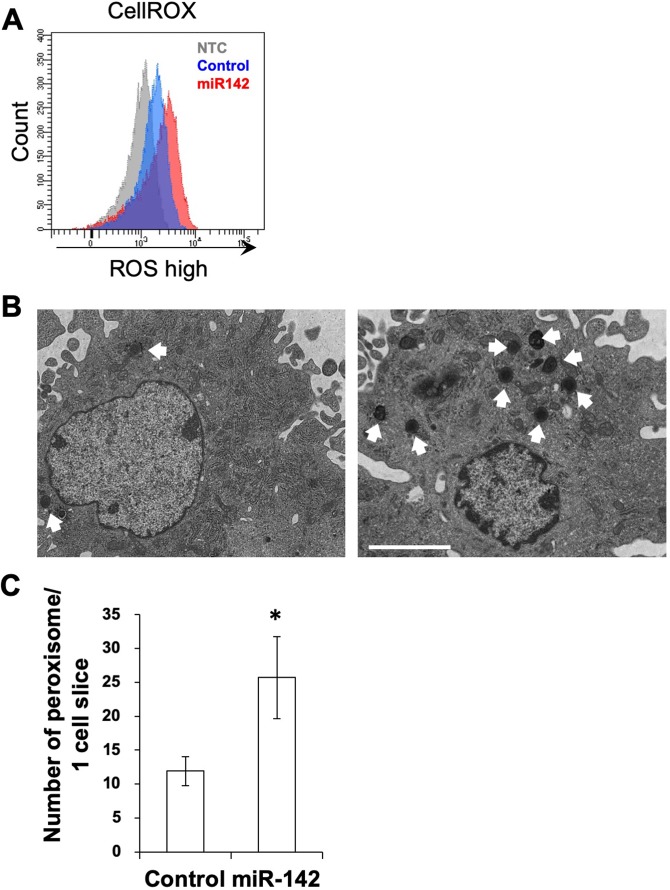
*miR-142* promotes ROS generation and peroxisome accumulation in BMMSCs *in vitro*. (**A**) ROS detection by the CellRox system in BMMSCs transfected with the *miR-142* mimic. (**B**) Transmission electron microscopic (TEM) image of BMMSCs transfected with the *miR-142* mimic. Left panel is TEM of BMMSCs expressing scrambled sequences, and right panel shows TEM of BMMSCs containing the *miR-142* mimic. White arrows show peroxisomes. Scale bar = 3 μm. (**C**) Number of peroxisomes per one cell slide (N = 10). Asterisk represents a significant difference compared with the control cells transfected with scrambled RNA at *P* < 0.05.

Next, we examined if *miR-142* was involved in ROS generation in BMMSCs. When BMMSCs were transfected with a *miR-142* mimic, ROS levels increased (Fig. 2A). To determine the reason for the increased ROS levels, we performed electron-microscopic observation of BMMSCs transfected with the *miR-142* mimic. Using TEM, we found that the number of peroxisomes increased in BMMSCs containing the *miR-142* mimic (Fig. 2B,C). From these results, we hypothesized that *miR-142* induced ROS generation via induction of peroxisome accumulation.

**Figure 3:**
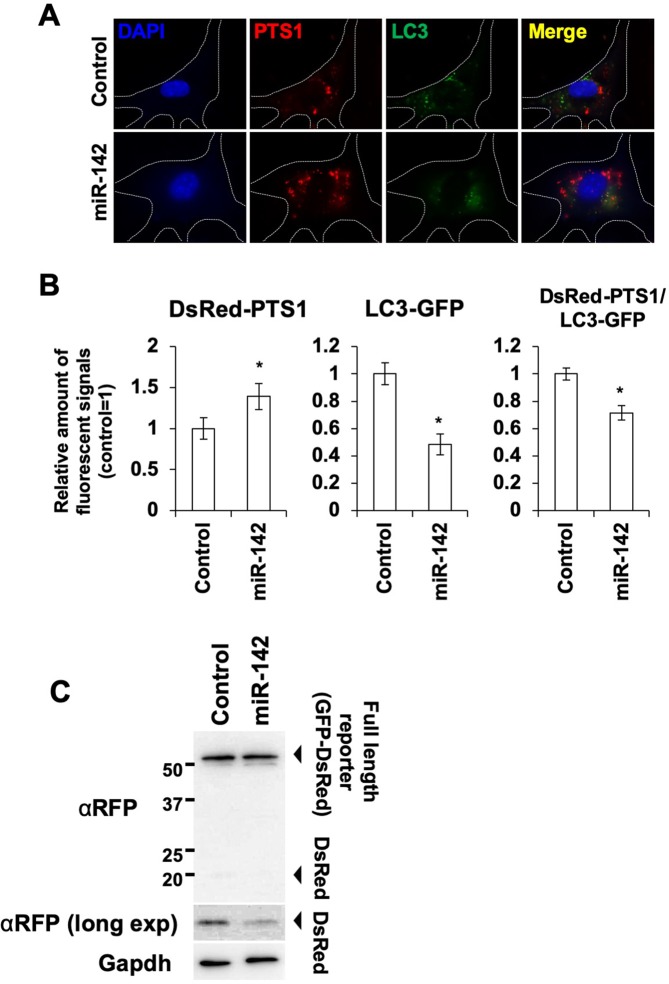
Selective autophagy for peroxisome is suppressed by *miR-142*. (**A**) Fluorescent imaging of pexophagy in BMMSCs transfected with *the miR-142* mimic. (**B**) Fluorescent imaging-based quantification of peroxisome and pexophagy in BMMSCs transfected with the *miR-142* mimic (N = 6). Asterisk represents a significant difference compared with the control at *P* < 0.05. (**C**) Detection of pexophagy activity using the PTS1-tagged GFP-DsRed tandem reporter. The GFP-DsRed is cleaved and the GFP is digested by lysosomal enzymes to yield the DsRed fragment. Decreased intensity of the DsRed band indicates the attenuation of pexophagy activity. αRFP (long exp) indicates the band obtained by prolonged exposure with the anti-RFP antibody (αRFP).

As a regulation mechanism of peroxisomal abundance, we focused on pexophagy, which is one of the most important regulatory mechanisms of peroxisomes. To detect peroxisome and pexophagic activity, we produced transgenic cells expressing peroxisome-targeted DsRed (DsRed-PTS1) and LC3B-GFP reflecting autophagy. Under normal conditions, approximately 33% of DsRed-positive peroxisomes overlapped with GFP fluorescence, indicating that these peroxisomes underwent pexophagy. When the cells were observed 1 h after rapamycin treatment, the abundance of GFP/DsRed-double-positive particles increased, while the DsRed single positive particles slightly decreased (Fig. S2). When the cells were treated with Wy-14643, which is a selective agonist of peroxisome proliferator-activated receptor-α^30^, the abundance of DsRed positive particles significantly increased (Fig. S2). Based on these results, we concluded that our system was useful for detecting both peroxisomes and pexophagy. By transfecting the *miR-142* mimic, the abundance of DsRed-positive particles representing peroxisomes significantly increased. However, GFP signals that represented autophagy were suppressed in the cells (Fig. 3A,B). To determine whether the pexophagy activity was affected by *miR-142*, we produced a pexophagy reporter that is a GFP and PTS1-tagged tandem monomeric DsRed (RFP). The reporter protein locates to the peroxisomes by the PTS1 peroxisome targeting signal, and when an autophagosome targets the peroxisome, the GFP domain of the reporter is digested by lysosomes and the DsRed fragment, which is relatively stable, remained^31^. Under autophagy-induced culture conditions, a clear band showing the DsRed domain was detected. Conversely, the DsRed fragments were decreased by transfection of *miR-142* mimic (Fig. 3C)

**Figure 4:**
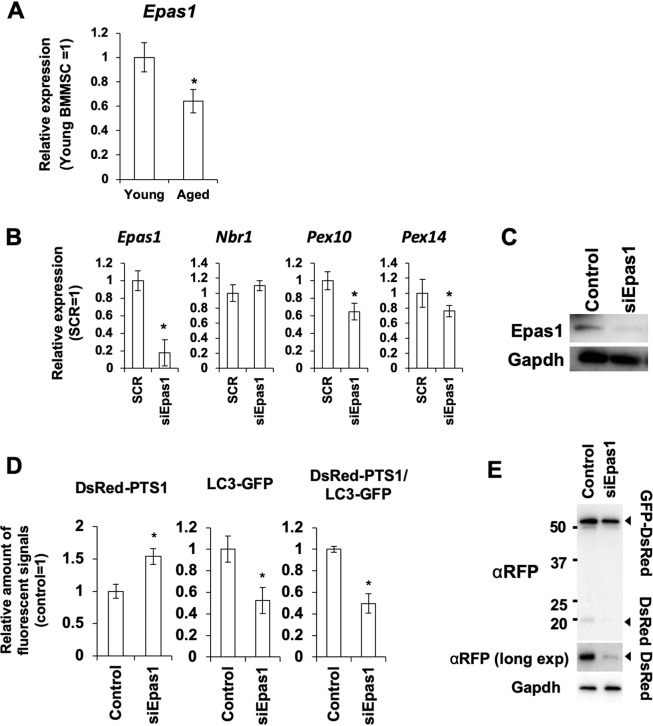
Epas1 is involved in regulation of pexophagy in BMMSCs. (**A**) Expression level of Epas1 mRNA in young and aged PαS fractions. Asterisk represents a significant difference between young and aged samples at *P* < 0.05. (**B**) Downregulation of Epas1 and the pexophagy-related genes *Nbr1*, *Pex10* and *Pex14* by siRNA (siEpas1) treatment in the BMMSCs (N = 6). SCR = BMMSCs treated with scrambled control RNA. Asterisk represents a significant difference compared with the control at *P* < 0.05. (**C**) WB analysis of Epas1 expression in the siEpas1-treated BMMSCs. (**D**) Fluorescent imaging-based quantification of peroxisomes and pexophagy in the BMMSCs transfected with siEpas1 (N = 6). Asterisk represents a significant difference compared with the control at *P* < 0.05. (**E**) Determination of pexophagy activity with the PTS1-tagged GFP-DsRed tandem reporter in BMMSCs transfected with siEpas1. αRFP (long exp) indicates the band obtained by prolonged exposure with the anti-RFP antibody (αRFP).

To elucidate the mechanism of the *miR-142*-mediated pexophagy regulation, we performed target prediction using miRDB^32^ and DIANA-Tarbase v 7.0^33^. From the results of the prediction analysis and previous studies^34^, we hypothesized that miR-142 affects peroxisome abundance through suppression of Epas1, which was an important regulator of pexophagy, in aged BMMSCs. To demonstrate that Epas1 plays an important role in pexophagy during aging, we observed the expression level of Epas1 in young and aged BMMSCs, and then performed siRNA-mediated suppression of Epas1 to evaluate pexophagy activity using the DsRed-PTS1/LC3-GFP system in BMMSCs. In the aged BMMSCs, Epas1 expression was suppressed compared with young BMMSCs (Fig. 4A). Treatment with siRNA against *Epas1* (siEpas1) resulted in approximately 85% suppression of *Epas1* mRNA compared with control treated with a scrambled siRNA sequence and ROS generation (Figs. 4B and S3). Western blot (WB) analysis showed that decreased Epas1 expression also occurred at the protein level with siRNA treatment (Fig. 4C). The decreased amount of Epas1 affected the pexophagy-related genes Pex10 and Pex14. Consistent with previous studies^34^, peroxisomal abundance increased and LC3-GFP-positive peroxisomes showing pexophagy decreased under siEpas1 conditions (Figs. 4D and S3). Consistently, the GFP-DsRed tandem reporter assay also shows that the reduction of Epas1 expression suppressed pexophagy activity (Fig. 4E).

**Figure 5:**
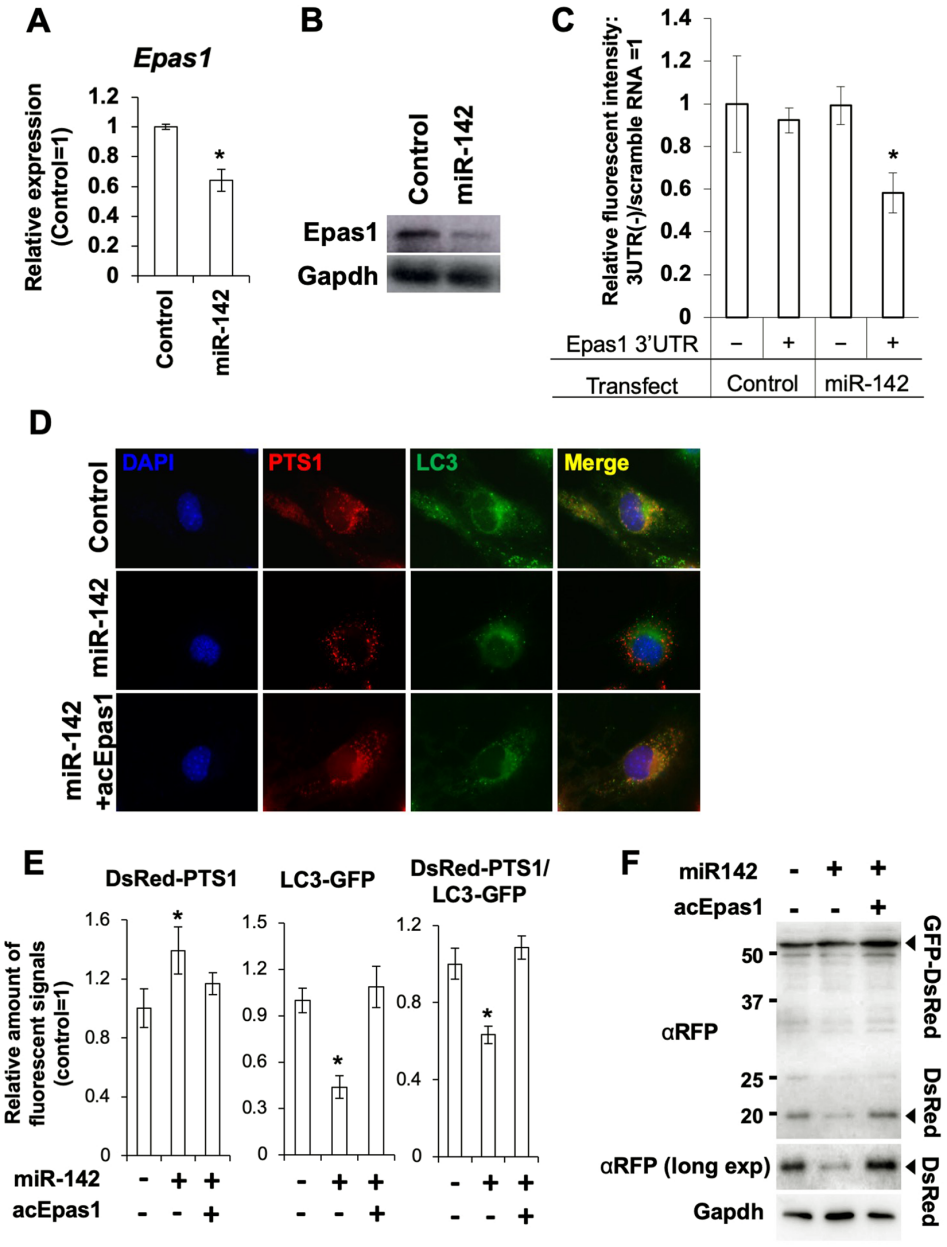
*miR-142* downregulates Epas1. (**A**) qRT-PCR of *Epas1* expression in BMMSCs treated with the *miR-142* mimic (N = 6). Asterisk represents a significant difference compared with the control at *P* < 0.05. (**B**) WB analysis of Epas1 expression in BMMSCs treated with scrambled control RNA (control) or with the *miR-142* mimic. (**C**) Luc assay with the Luc-Epas1 3′ UTR construct and the *miR-142* mimic (N = 3). Asterisk represents a significant difference compared with the control at *P* < 0.05. (**D**) Fluorescent imaging showing the results of the compensation experiment with the active form of Epas1 (acEpas1) in BMMSCs treated with the *miR-142* mimic. (**E**) Fluorescent imaging-based quantification of peroxisomes and pexophagy in BMMSCs transfected with the *miR-142* mimic, and both the *miR-142* mimic and the acEpas1 encoding plasmid (N = 6). Asterisk represents a significant difference compared with the control at *P* < 0.05. (**F**) Determination of pexophagy activity with the PTS1-tagged GFP-DsRed tandem reporter in BMMSCs transfected with the *miR-142* mimic and/or the acEpas1 encoding plasmid. αRFP (long exp) indicates the band obtained by prolonged the exposure with anti-RFP antibody (αRFP).

We investigated if *miR-142* downregulated Epas1 expression in BMMSCs. As expected, the expression level of *Epas1* was suppressed in BMMSCs transfected with the *miR-142* mimic (Fig. 5A). WB analysis also showed decreased expression of Epas1 in BMMSCs transfected with the *miR-142* mimic (Fig. 5B). To confirm if *miR-142* targets *Epas1*, we constructed a luciferase (Luc) expression plasmid containing the 3′ UTR sequence of *Epas1* and compared the expression levels between wild-type Luc and Luc-Epas1 3′ UTR in BMMSCs transfected with the *miR-142* mimic. In normal BMMSCs, the expression levels of Luc were not different either with or without Epas1 3′ UTR. In contrast, expression of Luc-Epas1 3′ UTR was significantly suppressed in BMMSCs transfected with the *miR-142* mimic (Fig. 5C). To further determine the molecular relationship between *miR-142*, Epas1, and pexophagy, we performed a compensation experiment using an overexpression plasmid for an active mutant of Epas1 (acEpas1), which is stable even under normoxia and lacks a 3′ UTR^35^. Consistent with our hypothesis, the introduction of acEpas1 restored pexophagy activity and suppressed the *miR-142*-induced peroxisomal accumulation (Fig. 5D,E), indicating that the *miR-142-*mediated suppression of pexophagy occurred via regulation of Epas1. To determine whether the suppression of pexophagy by miR-142 was canceled by overexpression of acEpas1, we performed the GFP-DsRed tandem reporter assay and observed that pexophagy activity was recovered by acEpas1 expression (Fig. 5F).

**Figure 6:**
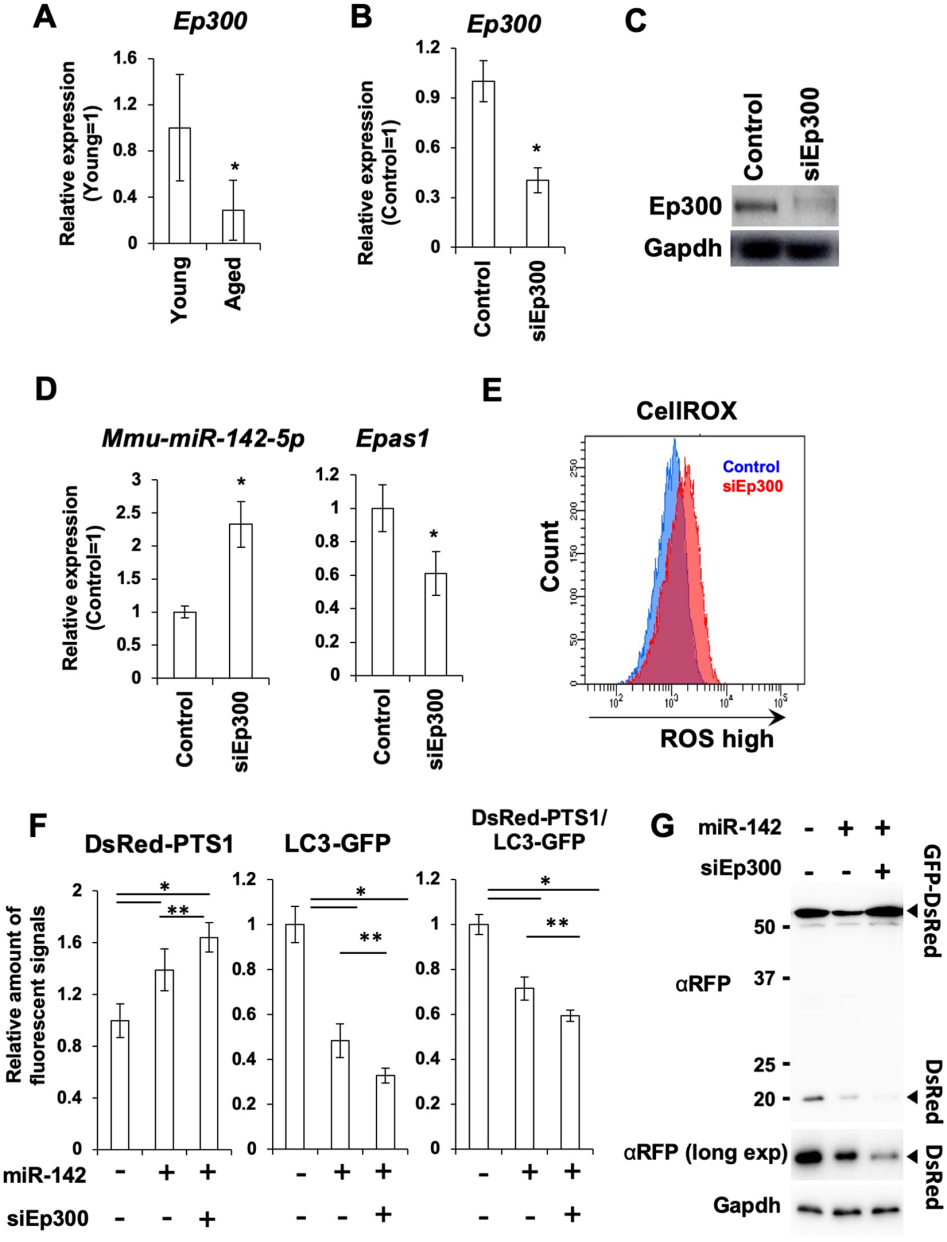
Downregulation of Ep300 leads to overexpression of miR-142 in BMMSCs. (**A**) qRT-PCR of *Ep300* in PαS-BMMSCs collected from young and aged mice (N = 6). Asterisk represents a significant difference compared with the PαS-BMMSCs from young mice at *P* < 0.05. (**B**) qRT-PCR of *Ep300* in BMMSCs treated with scrambled control RNA (control) or with siRNA against *Ep300* (siEp300) (N = 3). Asterisk represents a significant difference compared with the control at *P* < 0.05. (**C**) WB analysis of Ep300 expression in BMMSCs treated with scrambled control RNA (control) or with siEp300. (**D**) qRT-PCR of *miR-142* and *Epas1* in BMMSCs treated with siEp300 (N = 3). Asterisk represents a significant difference compared with the control at *P* < 0.05. (**E**) ROS detection by the CellROX system in BMMSCs transfected with siEp300. (**F**) Fluorescent imaging-based quantification of peroxisomes and pexophagy in BMMSCs transfected with the *miR-142* mimic and siEp300 (N = 6). Asterisk represents a significant difference compared with the control at *P* < 0.05. (**G**) Determination of pexophagy activity with the PTS1-tagged GFP-DsRed tandem reporter in BMMSCs transfected with the *miR-142* mimic and/or siEp300. αRFP (long exp) indicates the band obtained by prolonged exposure with the anti-RFP antibody (αRFP).

Next, we examined the molecular mechanisms responsible for upregulation of *miR-142* in aged tissues. It has been demonstrated that the expression level of *miR-142* is suppressed by acetyltransferase Ep300^36^. Ep300 is a protein that undergoes aging-associated downregulation^37^. We found that the expression level of *Ep300* was lower in aged BM and BMMSCs than in young mice (Figs. 6A and S1). From these data, we hypothesized that suppression of Ep300 increased expression of *miR-142* in aged BMMSCs. To validate this hypothesis, we suppressed expression of *Ep300* with a siRNA in BMMSCs and observed the expression levels of *miR-142* and *Epas1*. When the BMMSCs were treated with siRNA against *Ep300* (siEp300), a significant decrease in *Ep300* (Fig. 6B,C), an increase in *miR-142*, and a decrease in *Epas1* expression were observed (Fig. 6D). In the siEP300 treated cells, amounts of ROS levels, accumulation of peroxisomes, and amounts of pexophagy were examined. Transfection of siEP300 increased intra-cellular ROS levels (Fig. 6E). The effect of siEp300 and miR-142 was additive; both the fluorescence imaging assay with the DsRed-PTS1/LC3-GFP system and the GFP-DsRed tandem reporter assay showed that co-transfection of the miR-142 mimic and siEP300 increased suppression of pexophagy compared with the single transfection of miR-142 (Fig. 6F,G).

From these results, we concluded that *miR-142* received suppressive regulation by Ep300 and that disruption of Ep300 during aging could be a trigger for the upregulation of *miR-142* expression and the subsequent disruption of pexophagy regulation and ROS generation in aged cells.

## Discussion

Accumulated ROS disturbs the maintenance and proliferative ability of stem cells^38^ by activating cell cycle suppressors^39^ or inducing differentiation commitment^40,41^. Furthermore, elevated ROS levels in MSCs reduce their engraftment potential and induce apoptosis after transplantation^26,42^. Thus, elucidating the molecular mechanisms underlying ROS accumulation and the development of therapeutic ROS modulation are essential to address aging and aging-related diseases. As a molecule involved in ROS accumulation in aged stem cells, we focused on the aging-associated miRNAs and performed miRNA sequencing. The results revealed that miR-142 was significantly upregulated in aged BMMSCs.

Consistent with our observation, Park *et al*., also found that the expression level of *miR-142-5p* increases in dendritic cells derived from aged bone marrow^43^. However, Zang *et al*. reported that the expression level of *miR-142* in serum decreases during the aging process^44^. Tissue or cell lineage specificity could be responsible for the discrepancy. For example, Zang *et al*. observed decreased expression of *miR-29b* in serum obtained from aged individuals. However, *miR-29b* is upregulated in aged brain^45^, aorta^46^, and cochlear tissues^47^. These findings highlight that care is needed in the selection of *miR-142* as a marker for detection of tissue/organ aging or as a common molecule inducing stem cell aging in different tissues.

In the cultured BMMSCs transfected with *miR-142*, an increased ROS level was detected with the CellROX assay. Previously, several research groups have demonstrated the involvement of miRNA in ROS generation. For example, *miR-210*, which is upregulated during hypoxia, induces accumulation of ROS and apoptosis^48^ by suppressing mitochondrial activity^49^. *miR-142*^50^, *miR-377*^51^ and *miR-200c*^52^ are also reported to be involved in ROS generation by targeting anti-oxidant genes. However, suppression of antioxidants such as *Nrf2*, *Sod1*, *Foxo1*, and *Foxo3* by *miR-142* was not significant in the present study (Fig. S4). In a previous study, we addressed a mechanism of the aging-associated miRNA (Ag-miRNA) induced ROS generation and revealed that *miR-155* inhibits expression of antioxidant related genes and increased ROS levels by debilitating tolerance against oxidation stresses. We hypothesized that *miR-142* induced ROS accumulation through a considerably different mechanism from the previous models since overexpression of *miR-142* further exacerbated the miR-155-induced ROS accumulation (Fig. S5). Interestingly, TEM observation clearly revealed that the number of peroxisomes increased in BMMSCs transfected with the *miR-142* mimic. This phenomenon was reproducible even when unpassaged primary BMMSCs with short culture period (120 h) were used (Fig. S6).

Under normal physiological conditions, the amount of peroxisomes is regulated by a balance between biogenesis and turnover. Especially, the selective autophagy of peroxisomes—pexophagy—is an important process responsible for the maintenance of peroxisomal quality and quantity^53^. Senescent cells contain approximately twice as many peroxisomes immunolabeled with the peroxisomal membrane protein 70 kDa (PMP70) or Pex14p^20^, as compared to early passage cells. Similarly, peroxisomes proliferate in the human retinal epithelia of aged individuals^54^. These findings indicate that the turnover of peroxisomes is disrupted in aged cells/tissues, and could be a cause of ROS generation that occurs with aging. In our study with a transgenic BMMSC line stably expressing LC3-GFP/DsRed-PTS1, we hypothesized that *miR-142* suppressed pexophagy and led to increased numbers of peroxisomes. To date, it has been posited that a ubiquitin-binding autophagic receptor Nbr1 is necessary and sufficient for pexophagy activity^34,55^. On the other hand, miR-142 did not seem to affect the gene expression of Nbr1 at least in our study. Compared with the direct suppression of Nbr1 by siRNA, in which a three-fold increase in peroxisomes compared with control was detected, the effect of miR-142 overexpression for peroxisome accumulation was weak. However, the function of miR-142 was additive to siNbr1 treatment (Figs. S7 and S8). These findings indicate that miR-142 regulates pexophagy by targeting an upstream or independent molecule from Nbr1. We investigated the direct targets of *miR-142* using target prediction with open software, and focused on Epas1 (Hypoxia inducible factor 2A), which is a master regulator of the adaptive response to hypoxia. Walter *et al*. reported that hypoxia inducible factor (HIF) 2A ensures the efficient depletion of the peroxisome pool by simultaneously inducing pexophagy^34^. As a possible reason for the regulation of pexophagy by Epas1, peroxisomal function may be highly dependent on oxygen concentration^56^. Thus HIF signaling that is used to adapt to low oxygen conditions could be a suitable molecule to regulate the numbers of peroxisomes and their oxygen consumption. Although the aging-dependent expression changes of Epas1 in BMMSCs are still unclear, significant downregulation of *Epas1* in aged PαS BMMSCs were observed in the present study. Evidence supporting the idea that Epas1 was reduced in aged BM cells was also found in a previous gene expression profile^57^. These data prompted our hypothesis that Epas1 could be a promising candidate mediating *miR-142* expression and the dysregulation of pexophagy in aged BMMSCs. Consistent with a previous report^34^, suppression of Epas1 resulted in deterioration of pexophagy and accumulation of peroxisomes. Furthermore, consistent with our expectation, Epas1 expression was suppressed in BMMSCs transfected with the *miR-142* mimic. However, overexpression of Epas1 counteracted the *miR-142* induced proliferation of peroxisomes. From these results, we concluded that *miR-142* suppressed pexophagy via regulation of Epas1.

**Figure 7:**
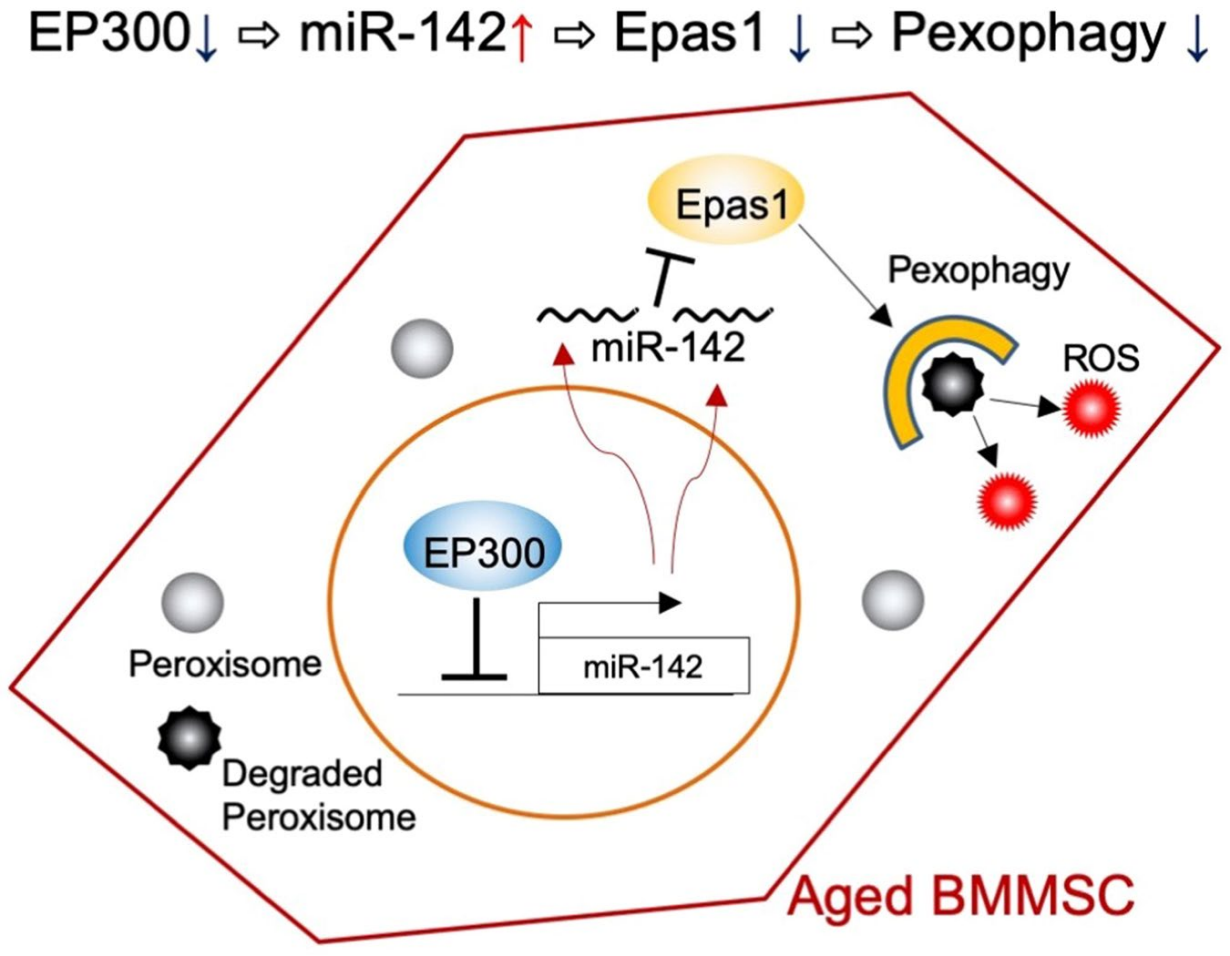
Schematic representation of miR142 regulation and function of the disruption of pexophagy and ROS generation. In normal (young) cells, the amount and quality of peroxisomes are maintained by pexophagy. In aged cells, the expression level of Ep300 that possesses a repressor molecule against miR-142 is downregulated. The upregulated miR-142 targets Epas1 and downregulation of pexophagic activity is induced.

As a factor triggering the upregulation of *miR-142*, we focused on Ep300. It has been reported that *miR-142* is downregulated by acetyltransferase Ep300^36^. Importantly, Ep300 appears to be downregulated with aging^37^ and with increasing doubling times in cultured cells^58^. Furthermore, it has also been reported that inhibition of Ep300 can induce aging phenotypes, such as senescence associated β-galactosidase expression^58^ and accumulation of DNA damage^59^. Consistent with our expectation, suppression of Ep300 led to increased expression of miR-142, accumulation of peroxisomes, and accumulation of ROS. These data support the conclusion that there is a molecular pathway that is responsible for inducing ROS generation in aged BMMSCs. In the first step of this pathway, reduction of Ep300, which occurs during aging or cellular senescence processes, leads to upregulation of *miR-142*. The increased *miR-142* targets *Epas1*, and the reduced level of Epas1 results in disruption of the normal maintenance of peroxisomes (Fig. 7) . From the perspective of a therapeutic application, *miR-142* could be an attractive target for miRNA-targeting therapy, since *miR-142* is highly expressed in Alzheimer’s disease^60^, multiple sclerosis^61^, and osteoporosis^62^, in addition to aged tissues. These findings indicated that the inhibition of *miR-142* using a specific inhibitor could be a potential therapeutic approach for some chronic diseases and various aging-associated cell/tissue degenerations.

Obviously, it is impossible to explain the aging-associated deterioration of peroxisomal maintenance and ROS generation only by the above scheme. We think there are at least three limitations. First, reduction of Ep300 and Epas1 may possibly generate ROS without mediation of *miR-142*. For example, Sun *et al*., reported that acetylation of Nrf2 by CBP/Ep300 involves maximum binding of Nrf2 to specific ARE-containing promoters^63^. This suggests that the reduction of Ep300 could lead to the wasting of anti-oxidation-related gene expression. Furthermore, several studies showed that depletion of Epas1 expression can reduce the expression of genes related to anti-oxidant systems, such as *Sod1*, *catalase*, and *heme oxygenase 1 (Hmox1)*^64,65^. Second, *miR-142* expression can be enhanced not only by reduction of Ep300 but also by stimulation with enhancer molecules. The inflammatory cytokines, IL-4 and IL-13, can trigger *miR-142* expression^66^. It is well known that aging tissues are in an inflammation-like condition and express various inflammatory molecules. Therefore, it is highly possible that these cytokine signals and Ep300 downregulation simultaneously enhance expression of *miR-142* in the actual tissues where aging-associated degeneration occurs. Third, we should pay attention to the roles of *miR-142* in normal development and tissue homeostasis. In general, miRNAs have multiple target genes. Thus, understanding its original role is very complicated. For example, even *miR-21*, which is one of the well-studied pathogenic miRNAs, is important for normal development^67,68^; it has been reported that *miR-142* is critical for hematopoiesis^69^ and the control of adaptive growth in cardiomyocytes^70^. Thus, further detailed studies of the targets and functions of *miR-142* in normal tissue homeostasis are essential. This understanding may enable the use of *miR-142* as a marker and target for therapeutic control.

## Materials and Methods

### Ethical statement

All procedures involving animals were approved by the Institutional Animal Care and Use Committee (IACUC) of Kindai University. All experiments using animals were performed in accordance with institutional guidelines and regulations. IACUC approved project No. is KAME-26-043.

### Isolation of the MSC from BM tissues (BMMSCs)

Four-week- and 1.5-year-old C57BL/6N male mice were used for the experiment as young and aged model. Bone marrow tissues were prepared as previously reported^71^. Long bones were collected from the hind limbs of the euthanized mice. The bone marrow was flushed out with α-MEM twice, and the residual long bones were cut into small pieces around 2–3 mm^3^ and treated with collagenase type II for 15 min. Then, dissociated tissues were washed twice with PBS and reacted with anti-PDGFRα (17-1401-81, eBioscience, San Diego, CA, USA), anti-Sca1 (61-5981-82, Thermo Fisher Scientific, Waltham, MA, USA), anti-CD45 (35-0451-U500, TONBO bioscience, San Diego, CA, USA), and anti-Ter119 (35-5921-U500, TONBO bioscience) for FACS isolation of the MSCs. As a control, cells were reacted with isotype IgG conjugated with each florescent dye (eBioscience). The PDGFRα+/Sca1+/CD45−/Ter119− population was sorted using a FACS Aria II (BD Biosciences, Franklin Lakes, NJ, USA).

### Detection of ROS in the BMMSCs from young and aged mice

BMMSCs were prepared as described above. ROS levels were analyzed using the CellROX Green Reagent (Thermo Fisher Scientific) and a FACS Canto^TM^ II (BD Biosciences).

### miRNA sequence

Libraries were generated from 10–20 ng of RNA, which was prepared from the FACS-sorted PDGFRα+/Sca1+/CD45−/Ter119− cells with the miRNeasy Micro Kit (Qiagen, Hilden, Germany), using the SMARTer smRNA-seq Kit (TAKARA Bio Inc., Shiga, Japan). The next-generation single-read sequencing was performed using the sequencer NextSeq500 (Illumina, San Diego, CA, USA) using the single-end 50 nt high output sequencing mode. The trimmed reads were mapped to the reference genome of mouse (GRCm38.p5) by STAR (version 2.6.1a) program. The sequence quality was assessed with FastQC software.

### Preparation of mouse BMMSCs

BMMSCs were isolated as previously reported^26^. BMMSCs were cultured in α-MEM (Wako, Tokyo, Japan) containing 10% fetal bovine serum (Hyclone, Logan, UT, USA) under 5% CO_2_ and 5% O_2_ at 37 °C.

### Fluorescent observation of pexophagy

A cDNA encoding the monomeric derivative of DsRed fluorescent protein was cloned into the piggybac cDNA expression vector with the PTS1 (serine-lysine-leucine) sequence^72^ using the In-Fusion HD Cloning Kit (TAKARA Bio Inc., Shiga, Japan).

The LC3-GFP cDNA was amplified from the pEGFP-LC3 plasmid (Addgene #21073) using the Tks Gflex DNA polymerase (TAKARA Bio Inc.) and cloned into the piggybac cDNA expression vector. PTS1-DsRed and pEGFP-LC3 was introduced using a CUY21 electroporator (NEPA Gene, Tokyo, Japan). Fluorescent images were captured using a BZ-X710 all-in-one fluorescent microscope (KEYENCE Corporation, Osaka, Japan) and analyzed integrated values of fluorescent brightness for each channel by an image analysis software BZ-X Analyzer Ver 1.3 (KEYENCE Corporation). To determine whether each fluorescent protein localized to peroxisomes and autophagosomes, a chemical activator of peroxisome proliferation, Wy14643^30^ and that of autophagy, Rapamycin was used^73^. DsRed/GFP double-positive dots were counted as peroxisomes processed by autophagy (pexophagy).

### Pexophagy activity detection assay

The EGFP sequence was amplified using Tks Gflex DNA polymerase and subcloned into the Nco I restriction enzyme site with a GGC short linker using the In-Fusion HD Cloning system. To enhance pexophagy activity, the BMMSCs were cultured in the amino acid-free DMEM for autophagy induction (Fujifilm Holdings Corporation, Tokyo, Japan) for 1 h before sampling. The full length and digested reporter molecules were detected using anti-RFP antibody (αRFP, M204-3, MBL, Nagoya, Japan) by WB as detailed below.

### RT-PCR for miRNA

RNA preparation and cDNA production were performed using the TRI Reagent® (Molecular Research Center, Inc., Cincinnati, OH, USA) and the miRNA cDNA Synthesis Kit with the Poly(A) Polymerase Tailing Kit (Applied Biological Materials Inc., Rochmond, BC, USA). Quantitative RT-PCR was performed using BrightGreen Express 2X qPCR MasterMix-No Dye (Applied Biological Materials Inc., BC, Canada) with a Thermal Cycler Dice® Real Time System at 95 °C for 30 s followed by 40 cycles at 95 °C for 5 s and 60 °C for 15 s. Expression quantity was evaluated by the ΔΔCt method (ΔΔCt = ΔCt_sample_ − ΔCt_control_). *U6 snRNA* was used as a control gene for normalization.

### RT-PCR for mRNA

RNA isolation and subsequent reverse-transcription were performed using TRI Reagent® and the PrimeScript® RT Master Mix Kit (TAKARA Bio Inc.). Quantitative real-time PCR was performed using Perfect real-time SYBR green II (TAKARA Bio Inc.) and a Thermal Cycler Dice® Real Time System. Expression quantity was evaluated by the ΔΔCt method using *Gapdh* as a control gene for normalization. To prevent contamination of genomic DNA, primers were designed to span at least one intron. Primer sequences are listed in Supplementary Table S2.

### WB analysis

All samples were lysed in SDS buffer, electroporated and blotted onto a PVDF membrane (Hybond-P; GE Healthcare Japan, Tokyo, Japan). The PVDF membranes were then treated with Block Ace for 1 hour (Dainippon Sumitomo Pharma, Osaka, Japan) and reacted with primary antibodies overnight at 4 °C. Chemiluminescence detection was performed with horseradish peroxidase (HRP)-conjugated secondary antibodies and Immunostar^®^ LD (Wako) reagents. Antibodies are listed in Supplementary Table S3. The raw WB data are shown in Supplementary Fig. S9.

### Transfection of the mimic RNA and siRNA into mouse BMMSCs

Mimic RNA of miR-142 (mmu-miR142a-5p mimic, GeneDesign, Inc., Osaka, Japan) and siRNA against *Epas1* or *Ep300* were transfected using the Lipofectamine™ RNAiMAX Transfection Reagent (Thermo Fisher Scientific). Sequences of the siRNAs used are shown in Supplementary Table S4.

### Transmission electron microscopy (TEM) observation

BMMSCs were fixed in 2% glutaraldehyde in 50 mM PIPES buffer at room temperature for 2.5 h, then post-fixed, following rinsing, in 1% OsO_4_ for 1 h. Following dehydration in ethanol, the cell pellets were embedded in epoxy resin, cut into 70 nm sections, stained with uranyl acetate and lead citrate, and viewed with a HT7700 Automated TEM (Hitachi High Technologies, Inc., Tokyo, Japan) at 100 kV.

### Statistical analysis

Significant differences were detected with the Tukey-Kramer HSD test when the experiment had >three different groups. The data from two different groups were analyzed with a Student’s t-test, as appropriate. Caliculations were performed using JMP software version 10.0.0 (SAS Institute, Cary, NC, USA). Differences were assessed with a two-sided test with an α level of 0.05. The numbers of replicates (N) indicate biological replicates from independent experiments.

## Supplementary information

Supplemental information.
