# Supplementary material for: miR-142 induces accumulation of reactive oxygen species (ROS) by inhibiting pexophagy in aged bone marrow mesenchymal stem cells
Source: Sci Rep. 2020 Feb 28;10:3735. doi: 10.1038/s41598-020-60346-2 (PMC7048811; doi:10.1038/s41598-020-60346-2)

Supplemental information

miR-142 induces accumulation of reactive oxygen species (ROS) by inhibiting pexophagy in aged bone marrow mesenchymal stem cells

Kei Houri[1], Tatsufumi Mori[2], Yuta Onodera[3], Takatoshi Tsujimoto[1], Toshiyuki Takehara[3], Shinichi Nakao[1], Takeshi Teramura[3], and Kanji Fukuda[3].

[1]Department of Anesthesiology, Kindai University Faculty of Medicine, Osaka, Japan

[2]Kindai University Life Science Research Institute, Kindai University, Osaka, Japan

[3]Division of Cell Biology for Regenerative Medicine, Institute of Advanced Clinical Medicine, Kindai University Faculty of Medicine, Osaka, Japan

Supplementary Table S1. miRNAs upregulated in aged BMMSCs

miRNAname	Mean	log2FoldChange	*P* value
Mir425	230.266081	3.135168058	0.007902471
Mir125b-1	186.117592	3.220966435	0.009985053
Mir214	3890.0811	3.258890073	0.007058683
Mir378a	299.773182	3.278779402	0.009609238
Snord43	501.77723	3.50734719	0.002341578
Mir149	313.075386	3.565249179	0.002199149
Mir1843a	63.8443984	3.629629393	0.00701143
Mir130a	1376.18089	3.677474167	0.008501255
Mir99a	1040.26936	3.740453054	0.005042907
Mir15a	245.040587	4.031567822	0.008698456
Mir185	321.76506	4.084954473	0.002684158
Mir187	35.8809432	4.131366462	0.004827414
Mir29a	12109.6175	4.197468603	0.00228035
Mir29c	1743.45348	4.348149238	0.002539963
Mir301	559.032301	4.460346607	0.002759419
Mir199a-2	76.6318106	4.546095953	0.00529546
Mir101b	186.39558	4.602071195	0.002003992
Mir34a	677.496041	4.687319844	0.000823224
Mir29b-1	41.5024625	4.815510491	0.002239933
Mir33	174.740708	4.85903694	0.000987964
Mir34c	36.2927761	4.913127231	0.004543989
Mir497	4238.95506	5.042763293	0.000511302
Mir223	1764.01424	5.070834192	1.18E-05
Mir101a	2212.51059	5.105676321	0.0006985
Mir199b	5810.05647	5.172824298	0.000356547
Mir147	82.7269378	5.386755835	0.000467494
Mir34b	52.1895268	5.419406201	0.000813319
Mir1247	20.2004047	5.618247038	0.001305936
**Mir142**	**4834.74345**	**6.441084435**	**6.47E-07**
Mir29b-2	129.047845	6.771142519	5.05E-05
Mir1941	126.391523	7.235640844	1.18E-05

Supplementary Table S2. Primer sequences used in this study

Gene	Forward	Reverse
Nbr1	AGAGGATCGTTGGAGTGC	AGGTCTCCGCAGCTTTAAC
Pex10	AGAGATCGAGTTGCTCTCGG	AGTGCAACCAGCGCTGCAC
Pex14	TGCCACAGCAGTGAAGTTCC	TGAAGGCTCGTCGGCAGCTG
Epas1	TCATGGTAGAATCTGACGAG	AGAAGGTCCAGTGGCTAGAG
Ep300	AGCAACCAACAGGAATGAC	ACACTGCCACGGATCATAG
Gapdh	TGGAGTCTACTGGTGTCTTC	TCTCGTGGTTCACACCCATC

Supplementary Table S3. Information on antibodies used in this study

Antibody	Company	Dilution
Hif-2 alpha (aa355-438, NB100-480)	NOVUS Biologicals	1/ 1,000 in Immuno-enhancer
Gapdh (FL-335, sc-25778)	Santa Cruz Biotechnology	1/ 5,000 in 0.2% Tween-TBS containing 10% Block Ace

Supplementary Table S4. Sequences of siRNA used in this study

Gene	Sense	Antisense
Ep300	CAAUAGAGCGGAAUACUAU	AUAGUAUUCCGCUCUAUUG
Epas1	GAAUUCACCCAAAAAUCU	UAGAUUUUUGGGUGAAUU

Supplementary Figure S1. Age-dependent expression changes in *miR-142*, *Epas1*, *Ep300* and ROS accumulation.

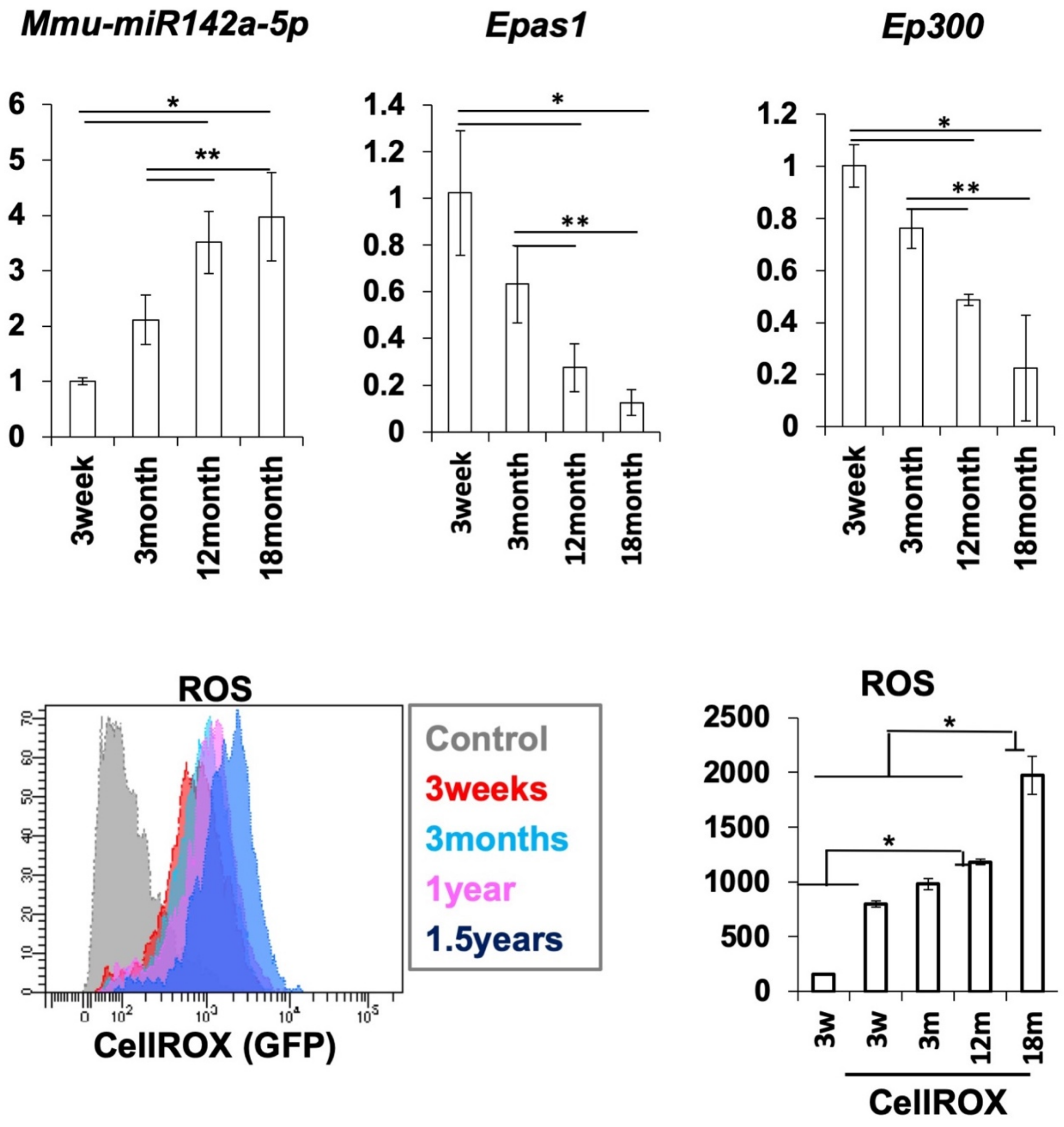

Supplementary Figure S2. Fluorescence imaging of peroxisomes using DsRed-PTS1 (peroxisome) and LC3-GFP (autophagy)

A, Fluorescent imaging of selective autophagy of peroxisomes (pexophagy) in normal BMMSCs, rapamycin treated BMMSCs, and Wy-14643 treated BMMSCs. The white dotted line represents the cell shape.

B, Fluorescent imaging-based quantification of peroxisome and pexophagy, as shown by the DsRed/GFP double-positive area for each experiment (N=6). The abbreviations Cont, Rap, and Wy represent control, rapamycin-treated, and Wy-14643-treated cells, respectively. An asterisk represents a significant difference compared with the control at *P*<0.05.

A

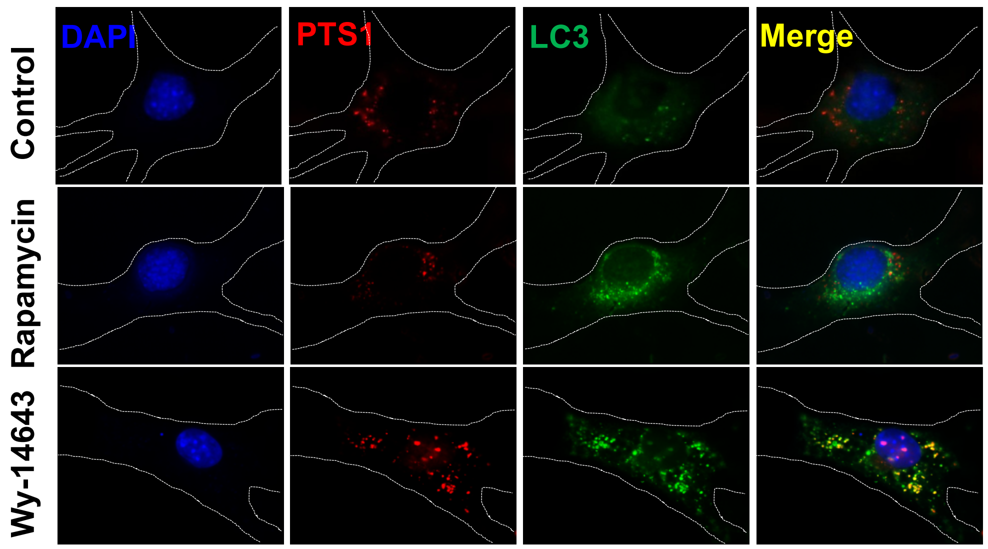

B

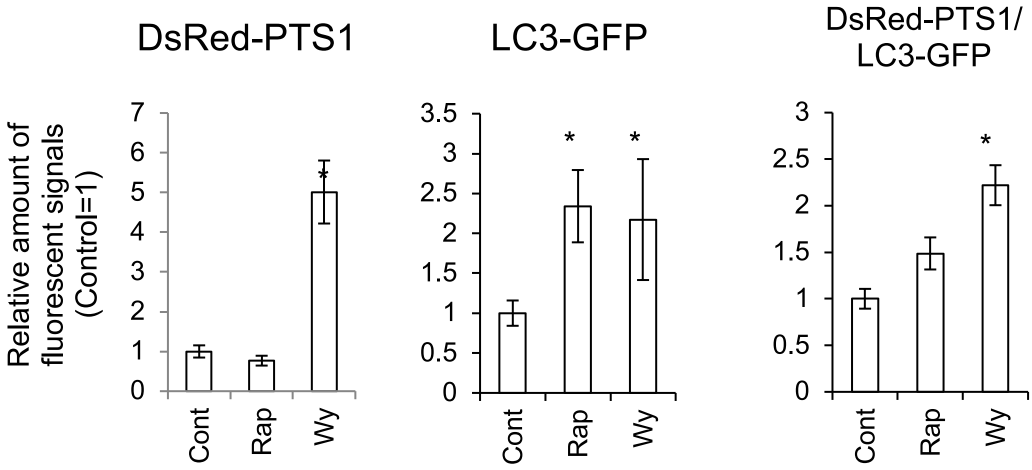

Supplementary Figure S3. Epas1 expression in aged BMMSCs

A, Detection of intra-cellular ROS by the CellROX assay. Suppression of Epas1 clearly increased ROS levels in the BMMSCs.

B, Fluorescent imaging of pexophagy in the BMMSCs transfected with siEpas1.

A

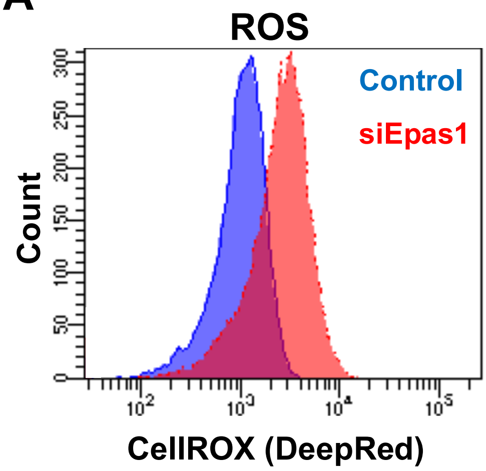

B

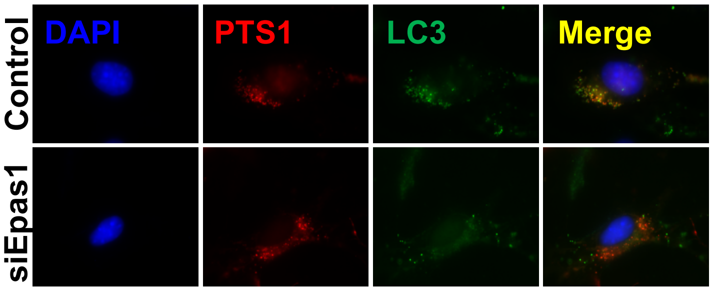

Supplementary Figure S4. Anti-oxidation related gene expression in siEpas1-treated BMMSCs

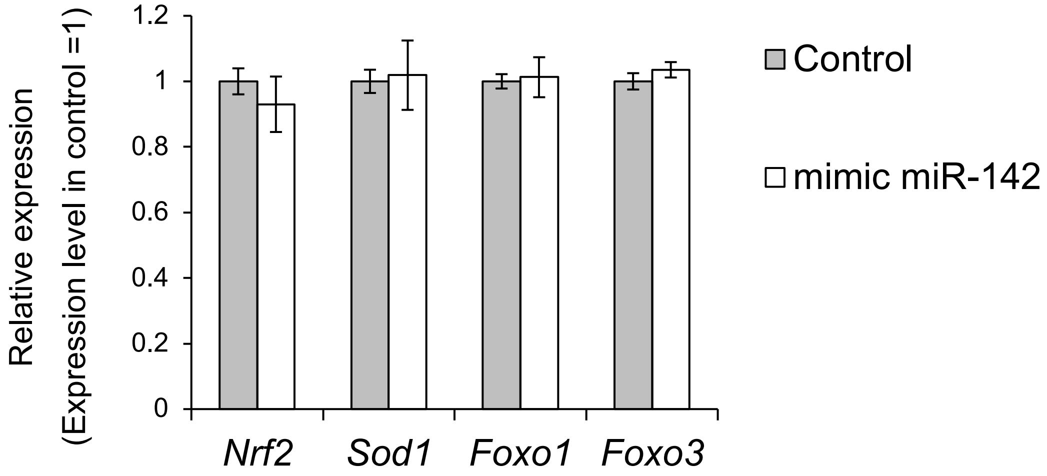

Supplementary Figure S5. miR-142 exacerbates miR155-induced ROS accumulation in BMMSCs

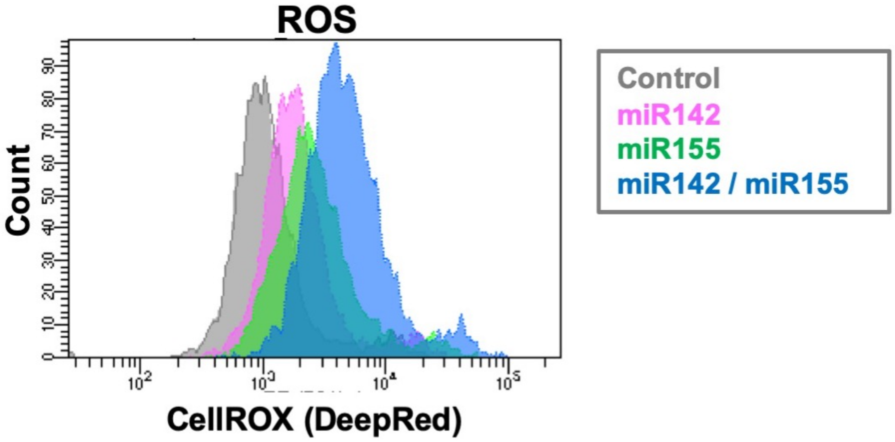

Supplementary Figure S6. miR-142 increases the number of peroxisomes in the BMMSCs cultured very short period.

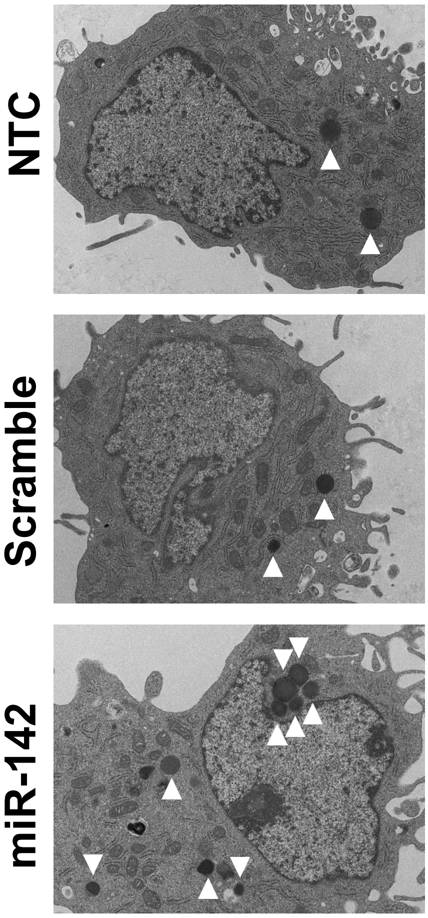

Supplementary Figure S7. Suppression of Nbr1 reduces pexophagy activity.

A, Fluorescent imaging of pexophagy in the BMMSCs transfected with siEpas1.

B, Fluorescent imaging-based quantification of peroxisome and pexophagy in BMMSCs transfected with the *miR-142* mimic and/or *Nbr1* siRNA (N=6). Asterisk represents a significant difference compared with the control at *P* < 0.05.

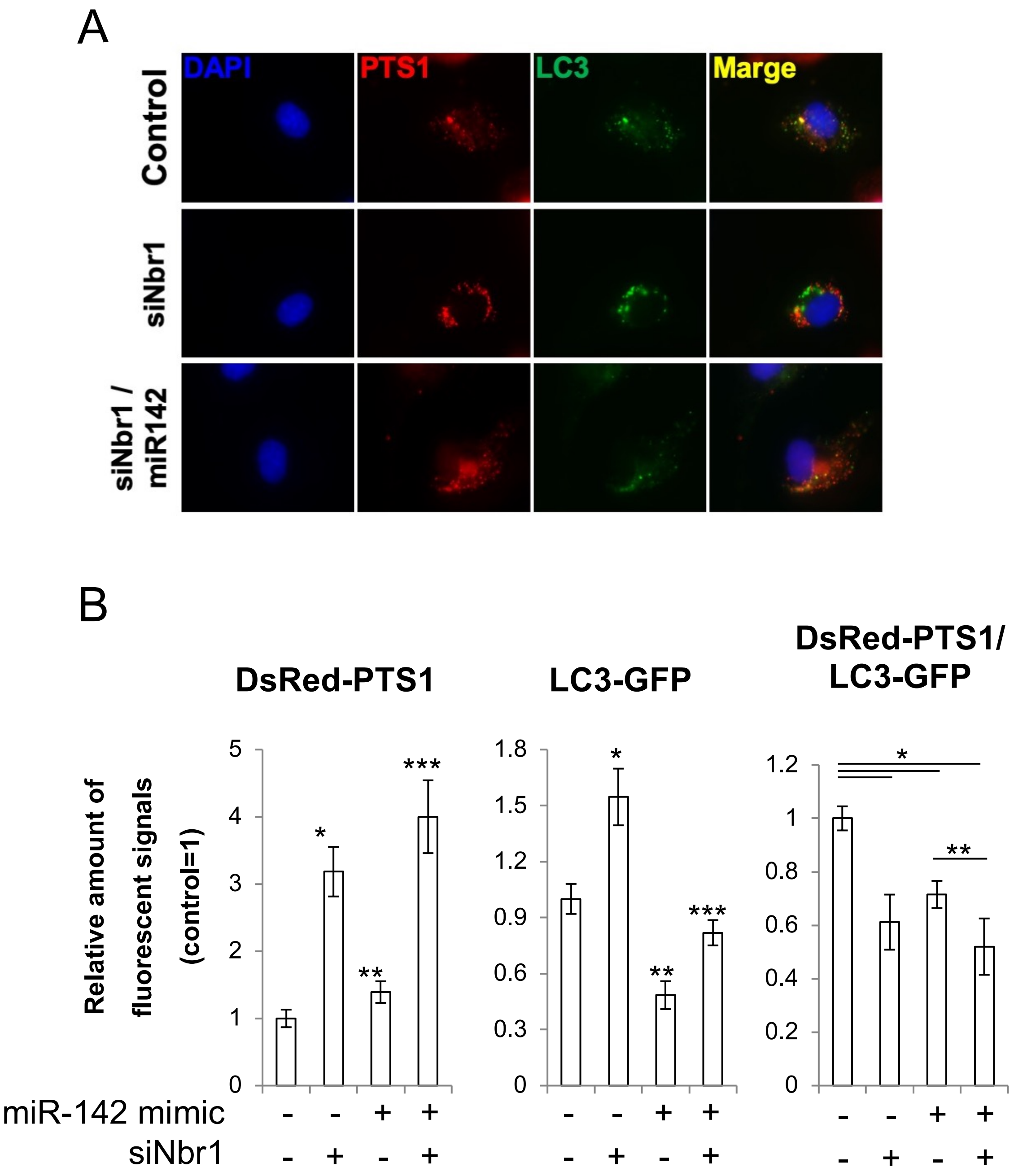

Supplementary Figure S8. Suppression of Nbr1 reduces pexophagy activity.

A, qRT-PCR for *Nbr1* in the miR-142 mimic transfected BMMSCs. NTC: non-treatment control, SCR: scrambled RNA, and mimic miR-142 transfected BMMSCs.

B, Pexophagy activity determined by the Pts1-tagged GFP-DsRed tandem reporter assay in each experimental group.

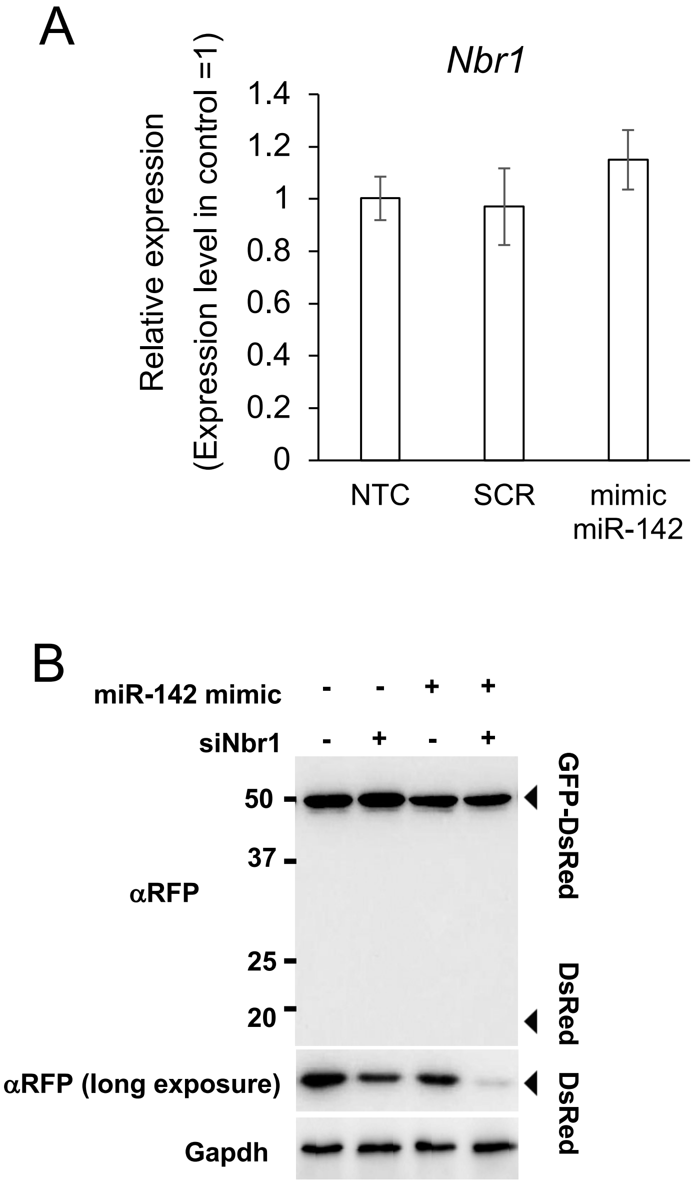

Supplementary Figure S9 . WB of Epas1, Ep300, and Gapdh in BMMSCs treated with siEpas1, the miR-142 mimic, and siEp300. Raw images of WB shown in Fig. 4B, Fig. 5B, and Fig. 6C. After protein transfer, the membranes, including objective proteins, were cut out and used for subsequent reactions. White-dotted lines represent the area shown in each figure.

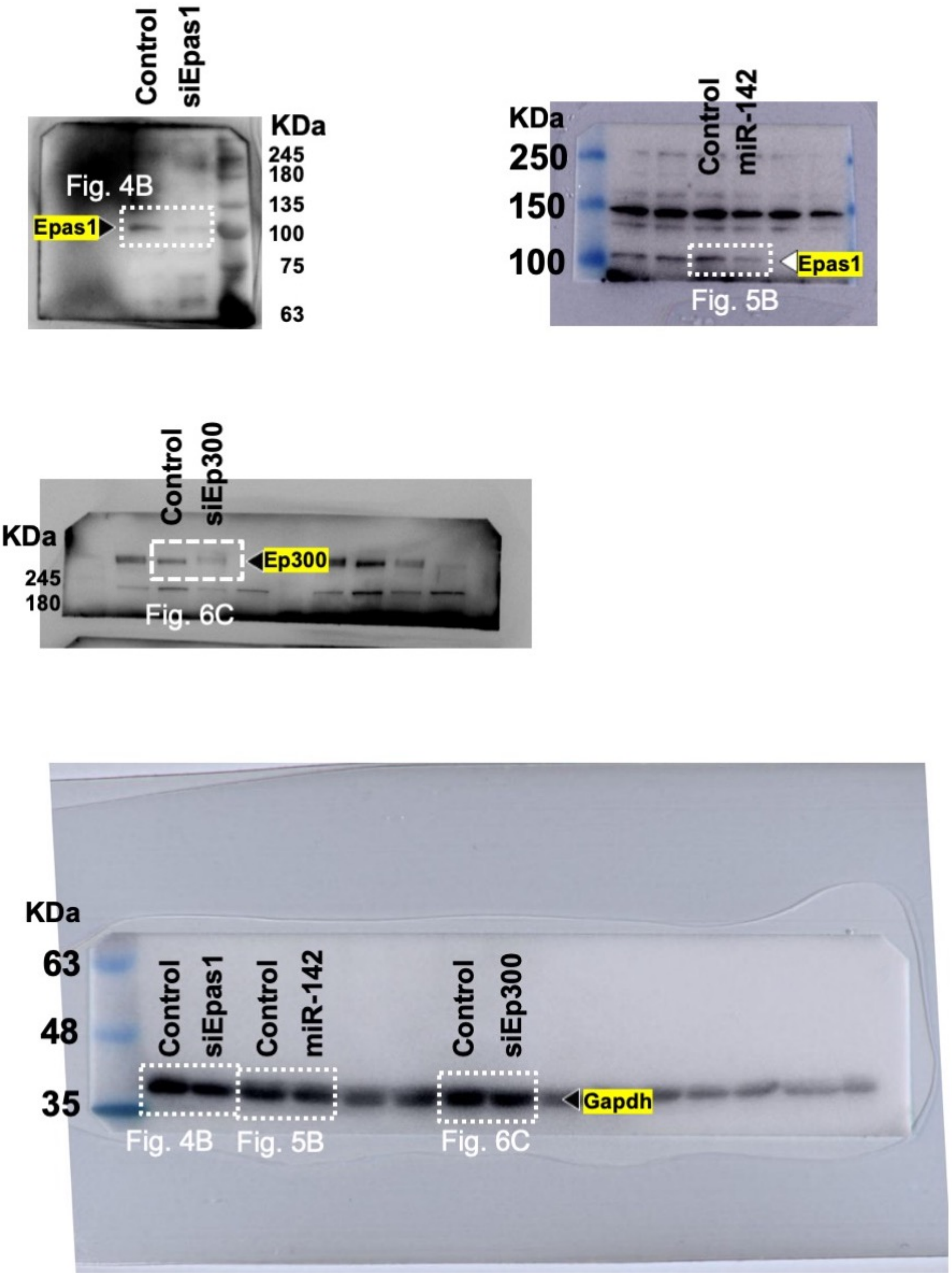

Supplement: Supplementary file 1 — Supplemental information. [file 41598_2020_60346_MOESM1_ESM.pdf]
